# Thermal energy storage – overview and specific insight into nitrate salts for sensible and latent heat storage

**DOI:** 10.3762/bjnano.6.154

**Published:** 2015-07-09

**Authors:** Nicole Pfleger, Thomas Bauer, Claudia Martin, Markus Eck, Antje Wörner

**Affiliations:** 1 German Aerospace Center (DLR), Pfaffenwaldring 38–40, 70569 Stuttgart, Germany; 2 German Aerospace Center (DLR), Linder Höhe, 51147 Köln, Germany

**Keywords:** eutectic mixture, molten salt, nitrate, phase change material, thermal decomposition

## Abstract

Thermal energy storage (TES) is capable to reduce the demand of conventional energy sources for two reasons: First, they prevent the mismatch between the energy supply and the power demand when generating electricity from renewable energy sources. Second, utilization of waste heat in industrial processes by thermal energy storage reduces the final energy consumption. This review focuses mainly on material aspects of alkali nitrate salts. They include thermal properties, thermal decomposition processes as well as a new method to develop optimized salt systems.

## Review

### Introduction

**Figure 1:**
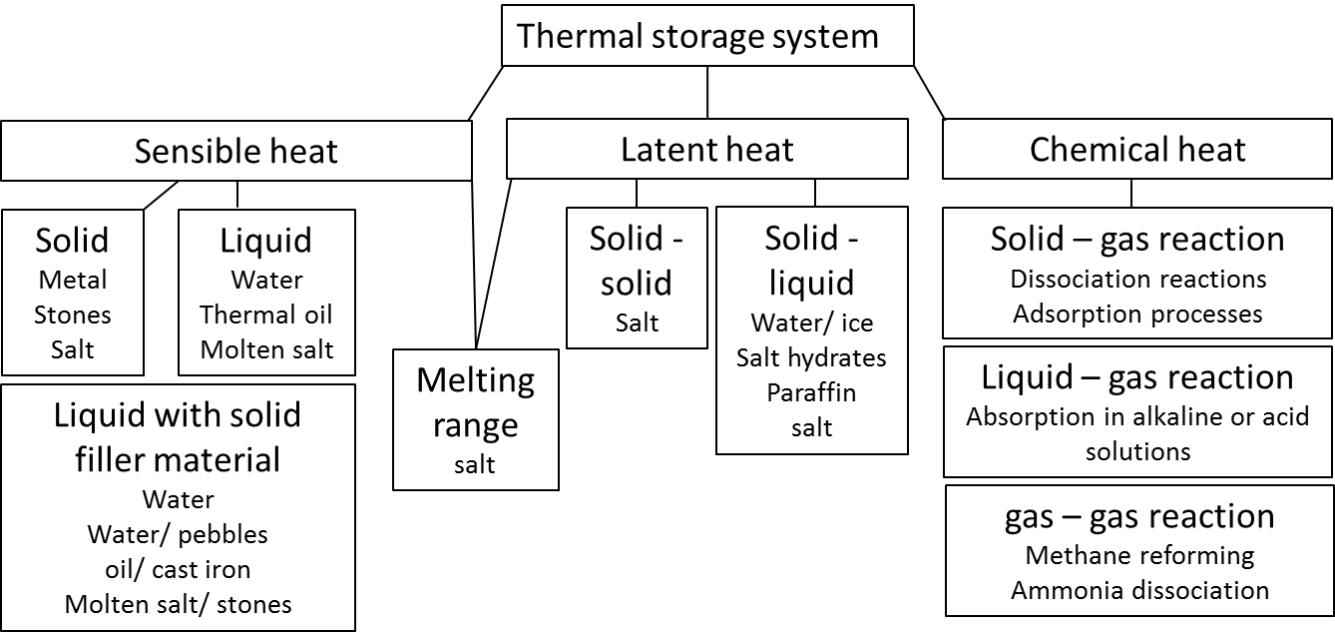
Classification of heat storage media.

Thermal energy storage (TES) is achieved by different techniques (Figure 1): sensible heat storage, latent heat storage and chemical heat storage.

The term “sensible heat” indicates that the storage process can be sensed by a change of the temperature. The relation of the change in temperature and the stored heat is given by the heat capacity *c*_p_.

In contrast to the storage of sensible heat latent heat cannot be sensed: The energy which is absorbed or released is stored by a phase transition which takes place at a constant temperature and therefore appears to be latent. Materials used for latent heat storage are called PCMs (phase change materials) because the heat storage is achieved by a phase change.

Another technique to store heat is thermochemical heat storage (TCS). TCS makes use of the enthalpy of reaction Δ*H*. In reactions featuring a positive change of Δ*H* (endothermic reaction) heat can be stored. The energy can be released by a backward reaction (Δ*H* < 0) afterwards.

Because of the possibility to store the compounds separately without the loss of energy thermochemical storage is appropriate for thermal energy storage over large period of times. TES is applied in the field of power generation, industrial process heat, space heating/ cooling as well as the management of thermal energy processes in vehicles. These classifications of storage characteristics and applications result in specific operation parameters and designs of TES systems.

Several TES media exist such as water, metals, ceramics, stones and salts. Table 1 gives an overview of sensible, latent and thermochemical TES processes using salts.

**Table 1:** Overview of salt application.

Temp. level	Salt type	TES type

<0 °C	Water–salt mixtures	PCM slurry
0–100 °C	Melting of salt hydrates in crystallization water	PCM
40–300 °C	Dehydration of salt hydrates	TCS
40–150 °C	Absorption in concentrated salt solutions	TCS
120–500 °C	Solid–liquid conversion in anhydrous salts	PCM
100–800 °C	Anhydrous molten salts	Sensible
100–800 °C	Anhydrous solid salts	Sensible
100–800 °C	Solid–solid conversion in anhydrous salts	PCM

The focus of this chapter is on salts in sensible and latent heat storage systems. Salt systems differ by important properties such as melting temperature and thermal stability which define the lower and upper limits of usable temperature in sensible heat storage systems. In latent storage systems the melting temperature defines the temperature at which the heat is stored. In thermal power plants the stored heat can be used to generate steam which drives turbines to produce electricity. Because the heat is generated at a specific and constant temperature and because of the temperature dependent water to steam transition the pressure of the steam can be adjusted to a level which is required by the turbine. Besides the melting temperature another important parameter for PCM applications is the melting enthalpy *H* (e.g., kJ·kg^−1^) which in addition to the material costs (e.g., €·kg^−1^) determine the specific material investment costs (e.g., €·kW^−1^·h^−1^). In case of sensible heat storage the specific material investment costs (e.g., €·kW^−1^·h^−1^) are defined by the material costs, the heat capacity *c*_p_ and the usable temperature range. The size of the sensible heat storage system is given by the product of the heat capacity and the density. The thermochemical properties depend on the ion system used. What concerns the anions the most important ions are nitrates, nitrate/nitrite mixtures, carbonates, chlorides, fluorides and carbonates. The cationic part of state of the art fluids usually consists of alkali/alkaline earth elements. The remainder of this chapter considers the respective materials more into detail.

### Sensible energy storage in anhydrous molten salts/nitrates

For sensible heat storage at elevated temperatures (*T* > 100 °C) molten salts are most suitable. Advantages of molten salts are the high thermal stability, relatively low material costs, high heat capacity, high density, non-flammability and low vapor pressure. Due to the low vapor pressure pressurized vessels are not required.

Compared to organic heat transfer fluids the melting point of molten salts is higher. Thus one major challenge with molten salts is to avoid freezing during operation. Hence, typically auxiliary heating systems or the development of salt formulations with low melting temperatures are required. A novel method to identify the composition of salt mixtures featuring a decreased melting temperature is presented at the end of this section.

Additionally limitations of molten salt storage may arise due to storage media costs, the risk of corrosion and the difficulty in hygroscopic salt handling.

For sensible heat storage in solar power plants, a non-eutectic molten salt mixture consisting of 60 wt % sodium nitrate (NaNO_3_) and 40 wt % potassium nitrate (KNO_3_) is used. This mixture is usually known as “Solar Salt”. Due to the increased amount of NaNO_3_ as compared to the eutectic mixture the material costs can be reduced. The non-eutectic mixture has a liquidus temperature of about 240 °C and the temperature limit of thermal stability is about 550 °C. For applications at higher temperatures salts with other anions, such as carbonates, chlorides and fluorides might be potential candidates. However experience with oxyanion salts and halogen salts is currently limited to theoretical studies [1–2].

#### Physico-chemical properties: thermal properties

**Figure 2:**
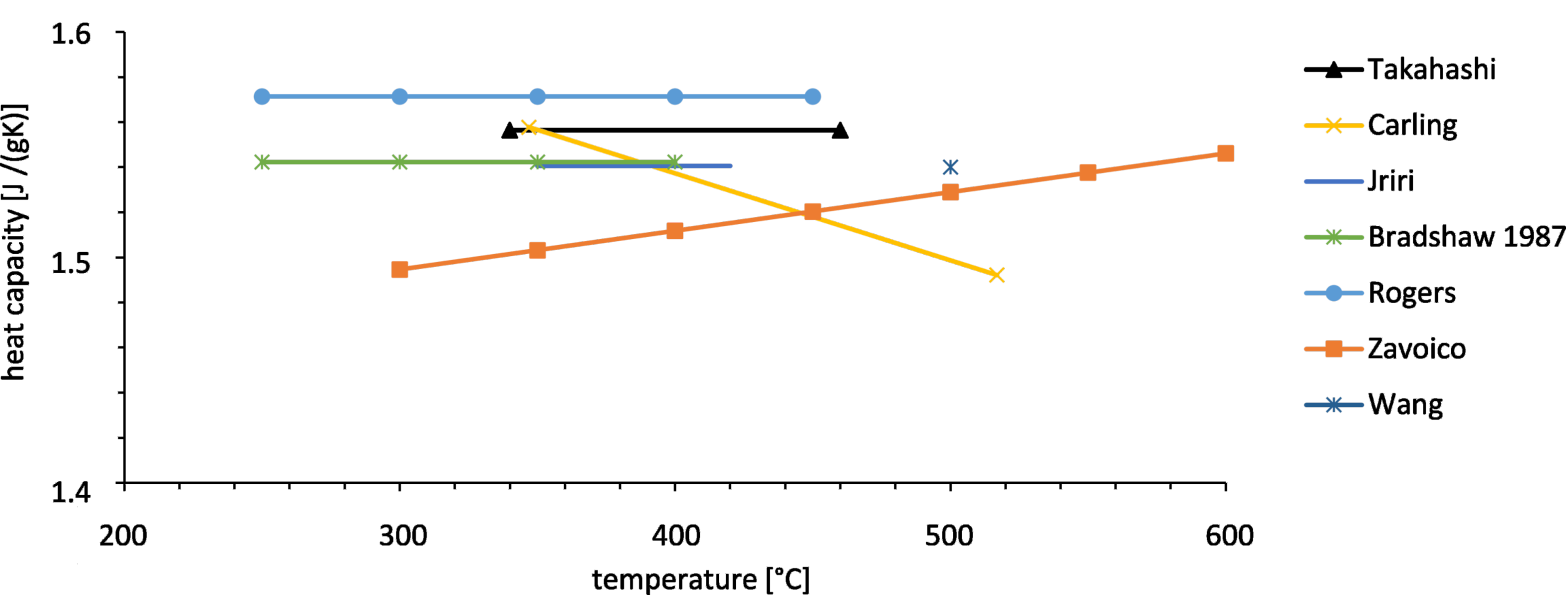
Heat capacity of Solar Salt in the liquid phase [3–9].

Characterization of thermal energy storage in molten salts requires data of salt properties in the liquid phase. For sensible storage media the storage capacity is directly proportional to the heat capacity which therefore is an essential parameter. Several data exist which are summarized in the following. The data show that the heat capacity is slightly increasing with temperature (see Figure 2).

**Figure 3:**
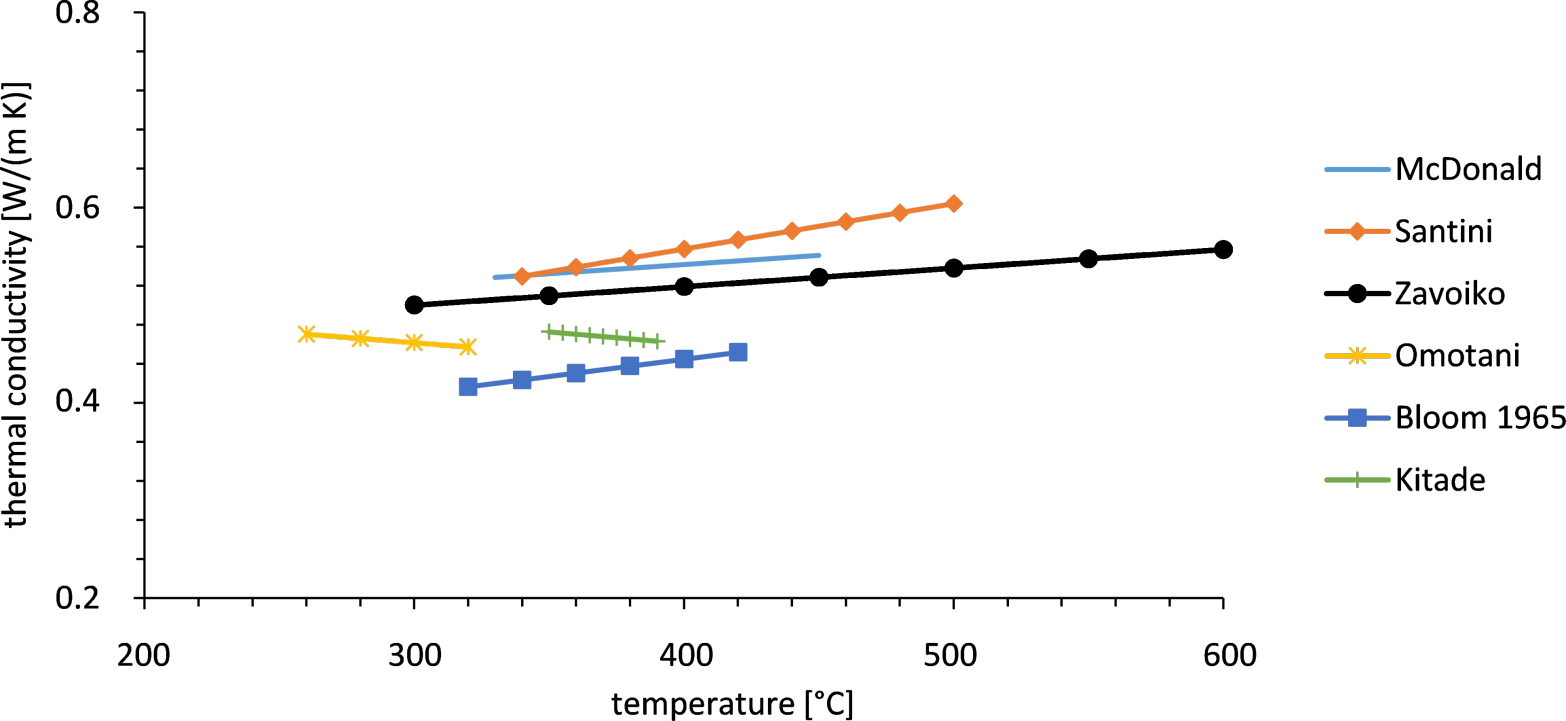
Thermal conductivity of Solar Salt reported by several groups [8,10–14].

Concerning the thermal conductivity several data exist which are not consistent and therefore rather give a rough idea, as shown in Figure 3. Even though the data differ in the different publications the measurements show that the thermal conductivity increases with temperature. More precise data require additional experiments.

**Figure 4:**
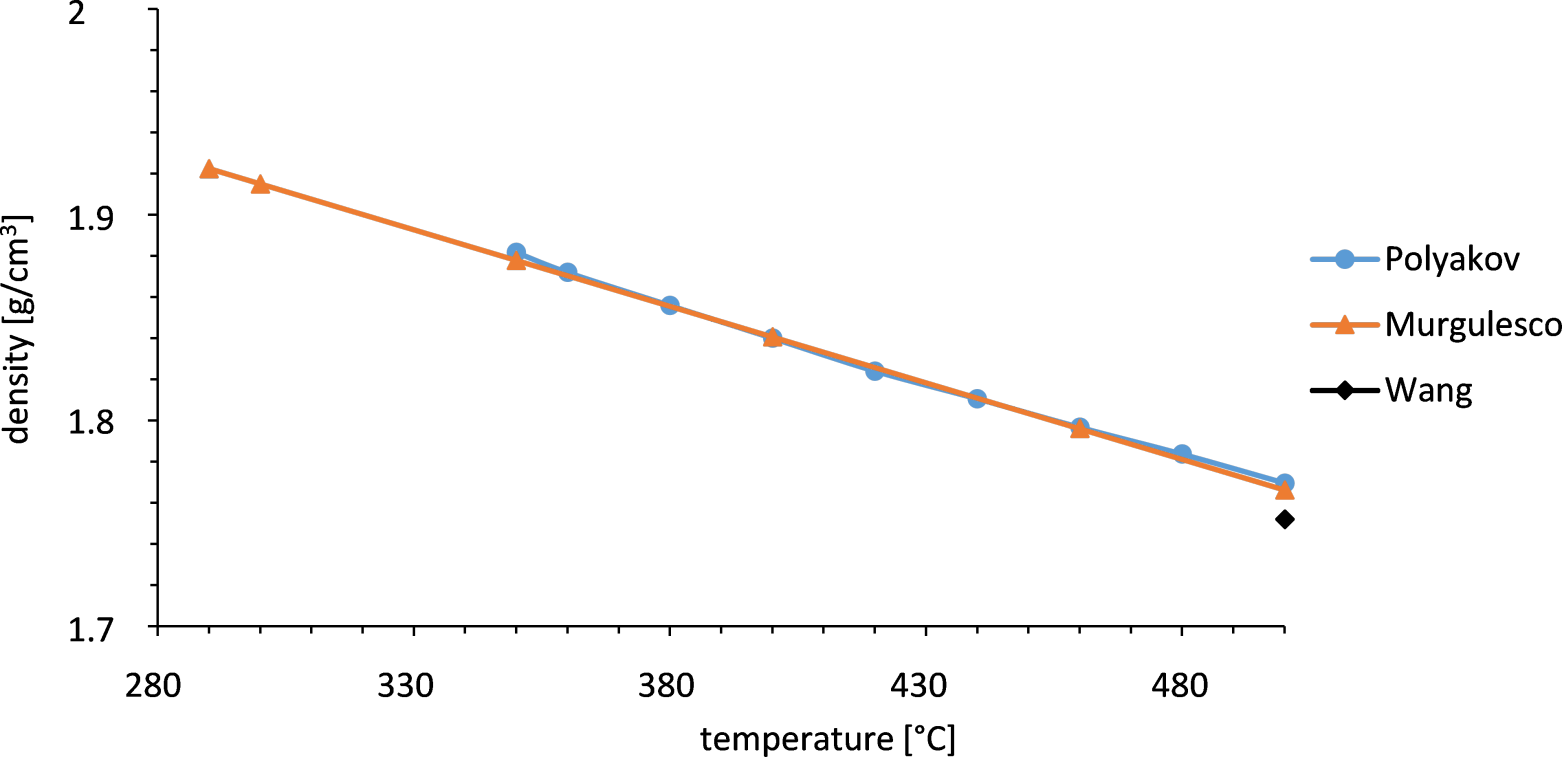
Density of Solar Salt in the liquid state [9,16–17].

As to the density there are consistent data from the literature in the liquid range. Also the density of multicomponent nitrate mixtures consisting of Ca(NO_3_)_2_, KNO_3_, LiNO_3_ and NaNO_3_ has been investigated [15]. It was shown that the temperature dependent molar volume can be estimated by a linear volumetric additivity rule based on the values of the individual constituents. However, only one literature source could be identified for the density in the solid range (Figure 4). Because accurate information of the salt property behavior in the solid-phase is necessary for recovery processes from a freeze event, the density of solid salt needs to be investigated further.

**Figure 5:**
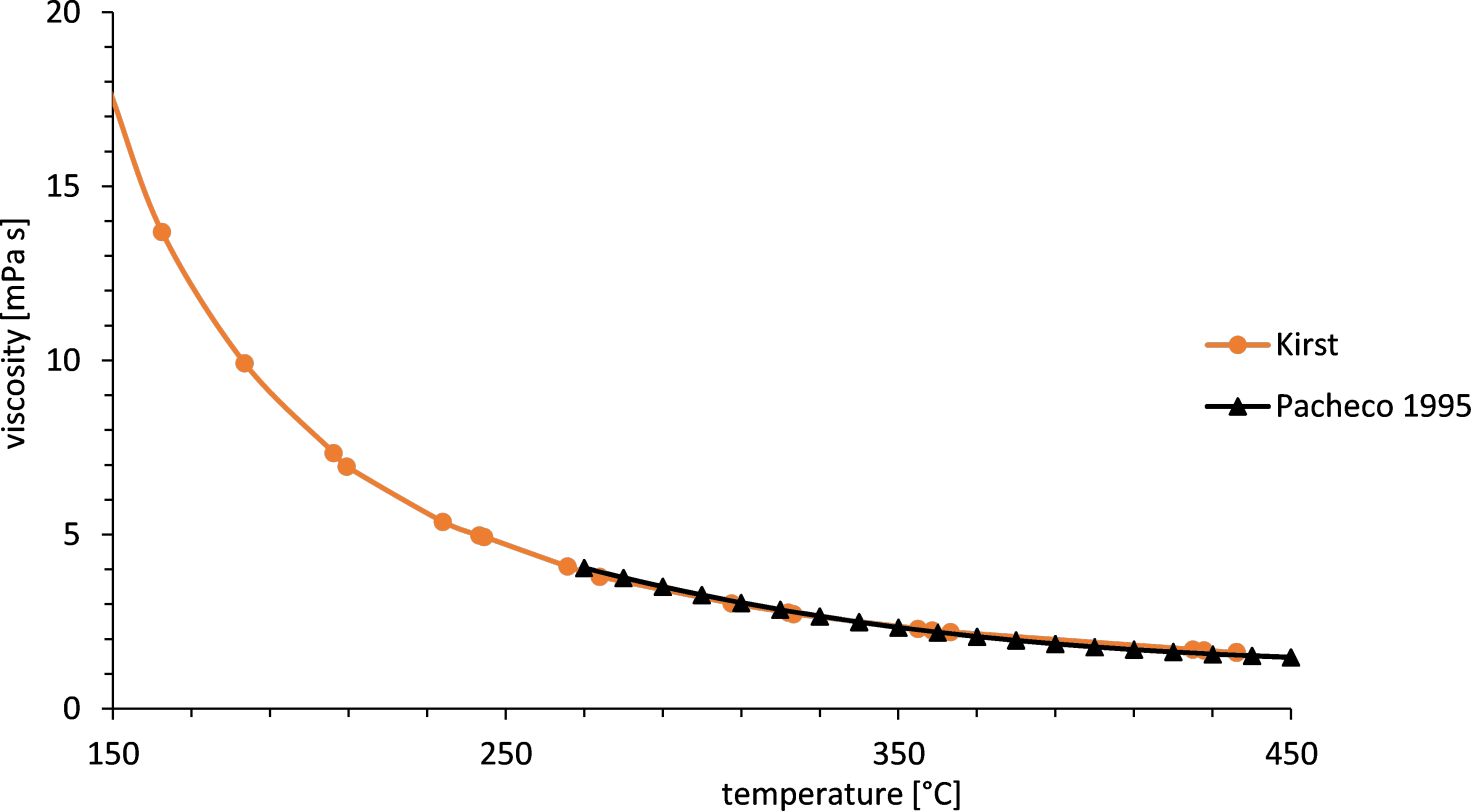
Viscosity of Solar Salt [18–19].

The viscosity is an important property for sensible storage media used in heat transfer applications with molten salt pumping. Figure 5 shows that the viscosity in the liquid range at 500 °C is in the same order of magnitude as the viscosity of water at ambient temperature.

#### Physico-chemical properties: thermal decomposition

The thermal decomposition of nitrate salts is a complex process which is dependent on the conditions, such as the cation composition, atmosphere, temperature and pressure. The cations have a significant effect on the thermal stability as their polarization power differs strongly. With increasing polarization power the distortion in the electron distribution in the anion is increased and thereby the stability of the salt is decreased [20]. Because the polarization power increases with the charge of the cation, the thermal stability decreases with the groups (columns) in the periodic table. Within one group the charge stays constant. Still the polarization power changes within one group because the second parameter affecting the polarization power is the radius. The higher the radius the lower is the polarization power. Because the radius is increasing with the period (rows) of the periodic table the stability increases within the group of the periodic table.

Thermal stabilities can be described by the temperature dependent equilibrium constant of decomposition reactions. Temperature dependent values are given for nitrates by Stern [21]:


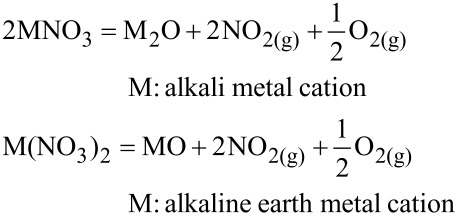


**Figure 6:**
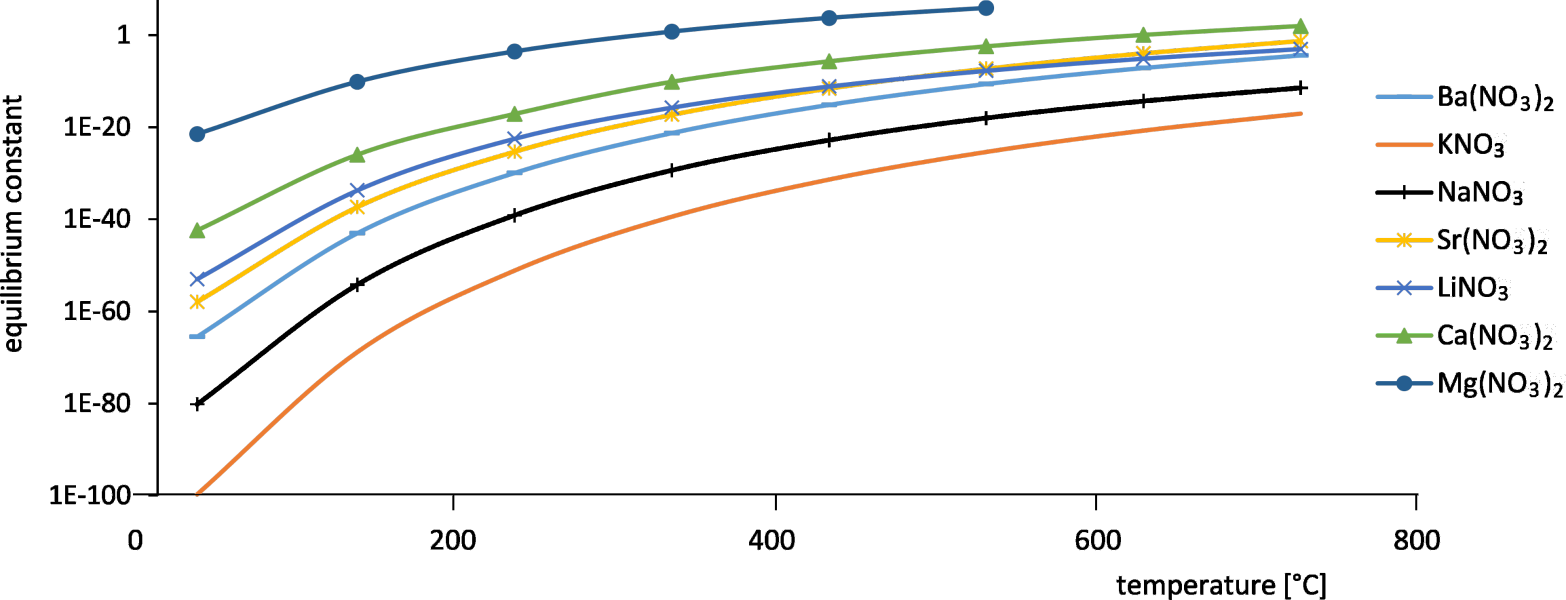
Temperature dependent equilibrium constant for alkali metal nitrates and alkaline metal nitrates.

Figure 6 shows the temperature dependent equilibrium constant *K*. For a value of *K* ≤ 10^−25^ the concentrations of the decomposition products are very low. Hence the salt can be considered stable.

**Figure 7:**
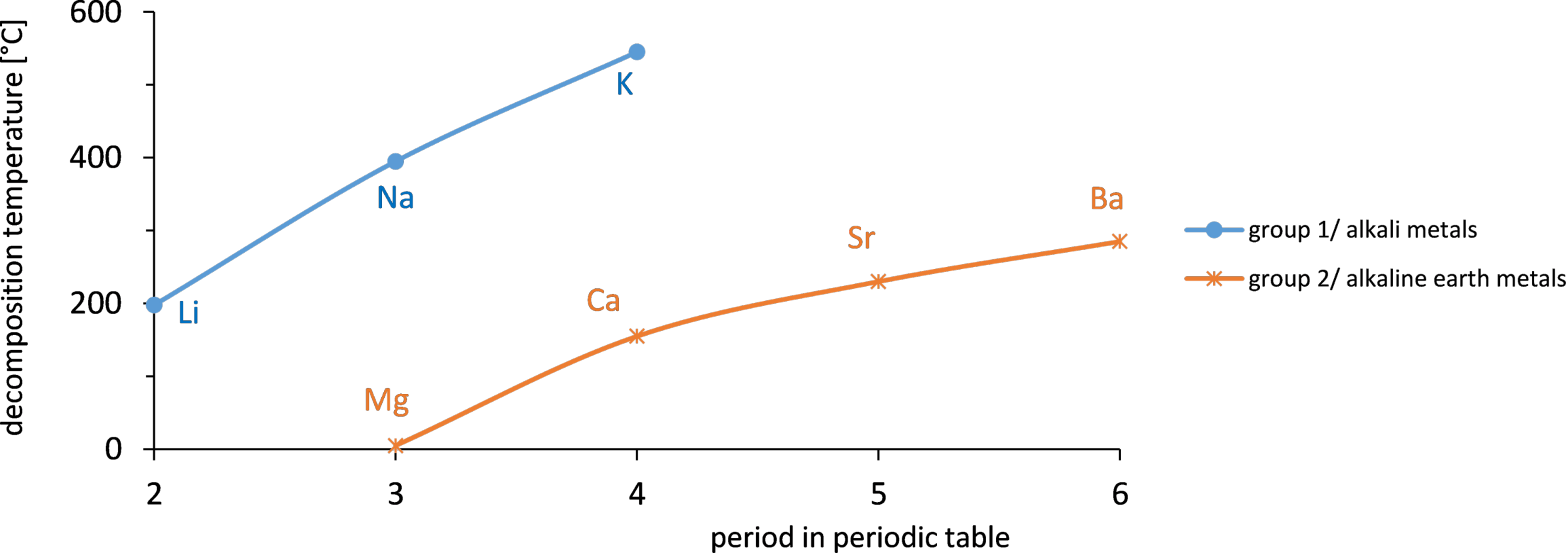
Relative decomposition temperature of nitrate vs position in the periodic table.

Figure 7 shows the decomposition with an equilibrium constant of *K* = 1 × 10^−25^ [21] versus the position of the elements in the periodic table (periods and the two groups alkali earth and alkaline earth metals). It can be shown that with increasing period in the periodic table the stability is increasing. The figure also shows that with increasing groups/charge of the cation the stability is decreasing. This is the reason why salts from groups > 2 are less suitable for heat storage application.

#### Development of new salt formulations with low melting point

The application of the state of the art sensible storage material “Solar Salt” is limited to processes with a lower operating temperature of 270 °C approximately. In particular parabolic through plants with molten salt as a heat transfer fluid in the solar field require mixtures with lower melting temperatures to avoid salt freezing and to simplify the solar field operation. Therefore salt formulations need to be developed with a reduced melting temperature. A new method has been presented by the authors in another article to develop new salt formulations [22] which is summarized in this section. Whereas compositions with low melting temperature have been identified by time consuming high-throughput experiments previously, the alternative method significantly reduces the number of experiments to identify the compositions of minimum melting mixtures.

**Figure 8:**
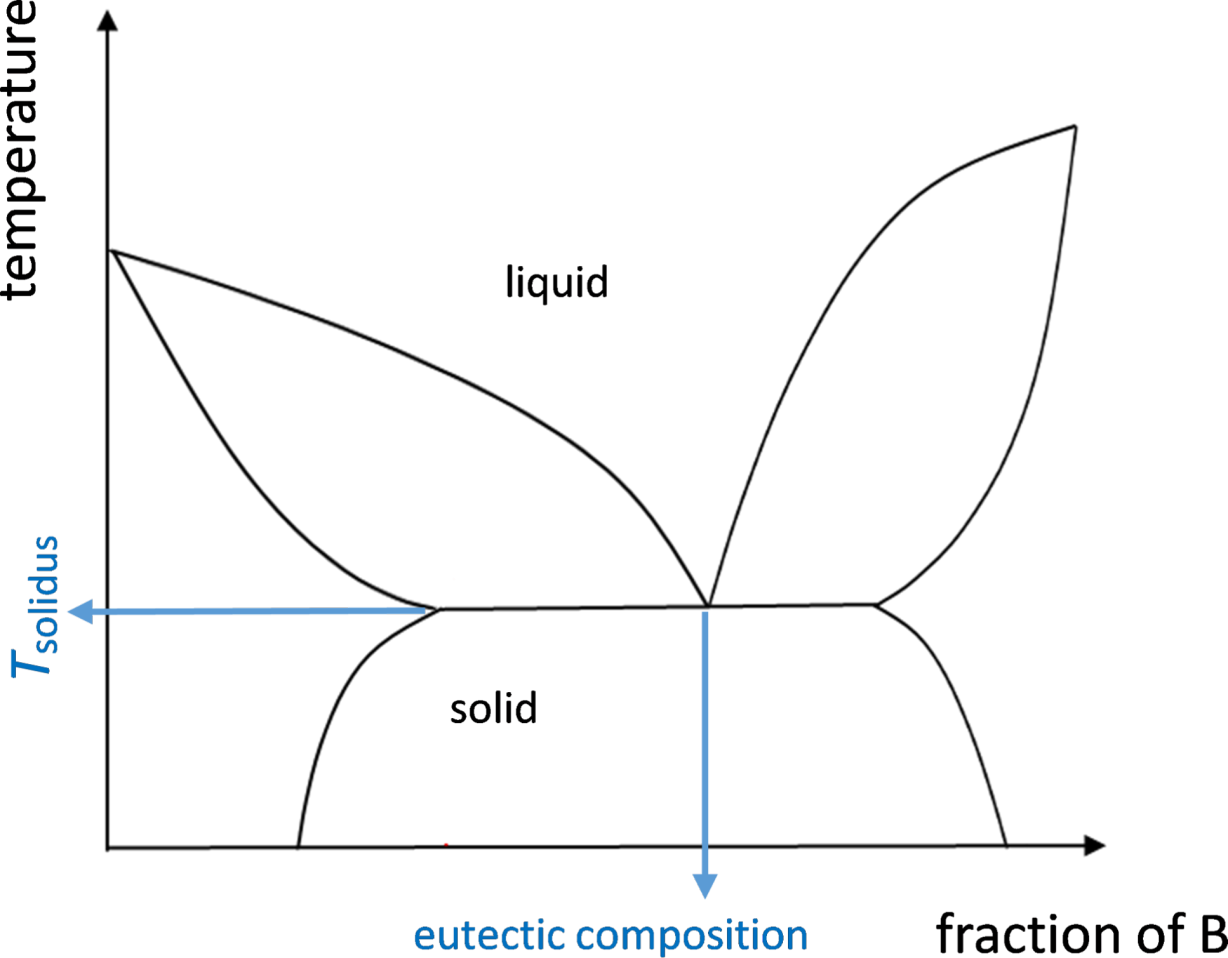
Scheme of phase diagram with eutectic mixture.

**Figure 9:**
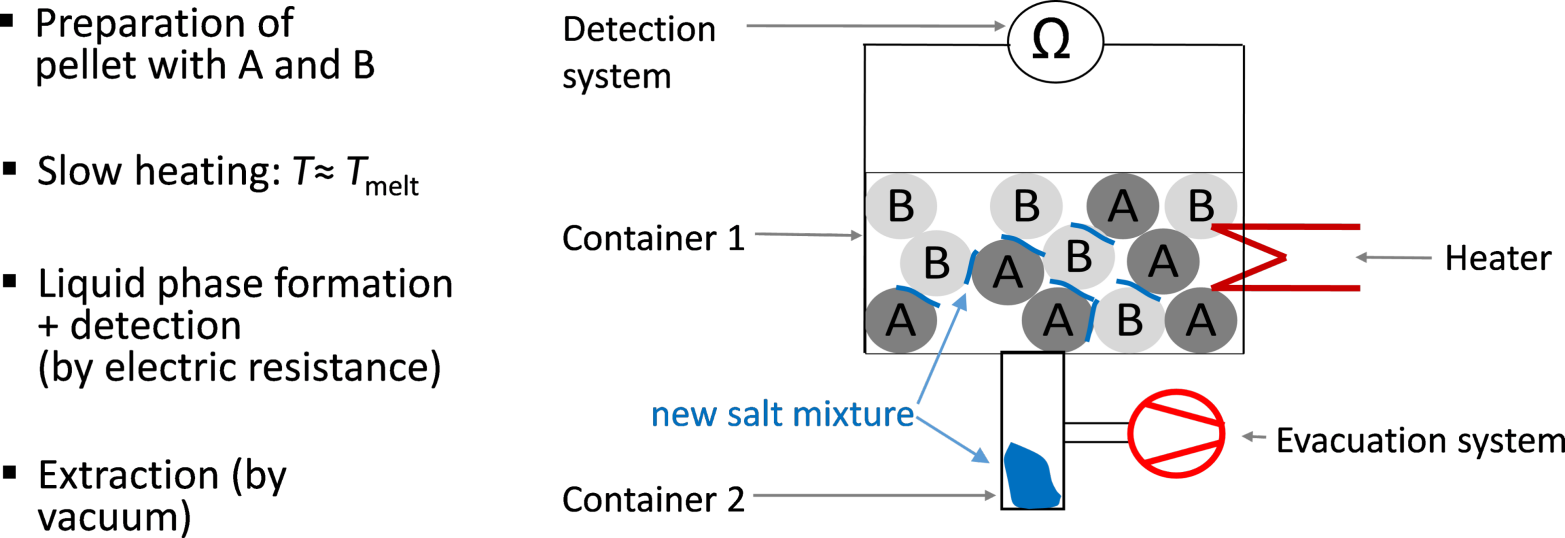
Schematic of the novel experimental method and apparatus to synthesize new salt mixtures.

The principle of the method is that the liquidus temperature of salts can be reduced by an increased number of ions. In other words, multicomponent salt mixtures can have lower liquidus temperatures as compared to simple binary or ternary salt systems. The liquid to solid phase transition of multicomponent salt mixtures is most conveniently obtained by inspection of the liquidus temperature in phase diagrams. The determination of phase diagrams however gets more challenging the more ionic species the salt mixture contains. Therefore the innovative method was developed to find salts with lower melting temperature without the need to fully determine phase diagrams. The method is based on liquid phase formation which is known from several processes: Eutectic bonding is a method to combine surfaces by eutectic alloy formation which occurs upon heating above the eutectic temperature. Similarly liquid phase sintering (LPS) is used in the field of high-temperature ceramics and metals. The innovative salt synthesis approach described in this section utilizes the liquid phase formation at the contact surface of different solid salts upon heating. The contact layer contains the eutectic composition. Figure 8 and Figure 9 illustrate the innovative salt synthesis approach.

A salt mixture of arbitrary non eutectic composition (for example X_non-eu_) is slowly heated above the solidus temperature *T*_solidus_. A measurement system detects the liquid phase above *T*_solidus_. The scheme of the phase diagram in Figure 8 shows that the molten salt composition is mainly the eutectic composition X_eu_ at the temperature *T*_max_. Following the detection of the liquid phase it is extracted via a filter and a valve. To find the composition of the salt system with reduced melting temperature, the extracted phase can be analyzed in terms of composition by analytical standard methods.

### Sensible energy storage in the liquid state with solid filler materials

The nitrate salts discussed in the previous sections are state of the art materials for the two tank concept. In the two tank concept two containers exist, referred to as “hot tank” and as “cold tank”. The heat is stored when pumping cold salt from the “cold tank” via a heat exchanger - providing heat by a heat transfer fluid - into the “hot tank”. The heat is recovered by the heat transfer fluid when hot salt is pumped from the “hot tank” to the “cold tank” via the heat exchanger. A drawback of this concept is the costs for the two tanks. In order to reduce the costs a lot of research was performed to find alternative storage concepts using other storage materials like concrete [23] or using a single-tank molten salt concept [24]. The single-tank molten salt concept provides a single storage tank by using the different densities of the cold and hot molten salt caused by the thermal gradient. Additionally cost reductions can result from partially replacing the molten salt storage material by low-cost filler material. This thermocline concept is described by [24]. Filler materials need to meet the following criteria:

- Inexpensive and widely available
- High heat capacity
- Low void fraction
- Compatibility with the heat storage materials such as nitrate salts
- Non-hazardous

A thermocline system with low-cost materials has the potential to reduce costs as compared to a two-tank molten salt storage system. The material selection of the filler material was supported by a geologist and a nitrate salt expert. Various natural stones were tested in Hitec XL^®^ (43 wt % KNO_3_ + 42 wt % Ca(NO_3_)_2_ + 15 wt % NaNO_3_) and in Solar Salt (60 wt % NaNO_3_ + 40 wt % KNO_3_) with a maximum temperature of 400 °C and maximum exposure duration of 1000 h in Hitec XL^®^ or 400 h in Solar Salt. The filler materials quartzite rocks and silica filter sand did not show any decomposition after 500 thermal cycles. Another advantage is the high availability.

The investigations were continued by Brosseau [25]. They focused on quartzite rock. Isothermal tests with a test-duration of one year were realized at temperatures of 450 and 500 °C. Additionally, 10 000 thermal cycles in the temperature range between 285 to 450 °C were performed. The heat transfer medium Hitec XL^®^ was used. The material tests of the quartzite rocks as well as sand were successful, apart from the observed calcium carbonate crust formation in the high temperature tests. Implementations in commercial-scale solar power plants do not exist so far because of concerns due to the calcium carbonate crust formation and its treatment in a large scale thermal storage unit.

The stability of the filler material is influenced by the molten salt (Solar Salt, HITEC XL^®^, etc.) and by the maximum operation temperature. In the recent years, material investigations were performed to reduce costs of the one-tank thermocline concept further by using filler materials with very low costs or improved material properties such as a higher heat capacity. One optional filler material is a very inexpensive material called Cofalit^®^. Cofalit^®^ is manufactured by the INERTAM Company in France and is produced by high-temperature plasma treatment (1500 °C) of asbestos-containing waste called ACW. Cofalit^®^ is a calcium magnesium iron alumina-silicate [26]. The thermophysical properties meet the required criteria of potential filler materials. It has a density of 3120 kg·m^−3^ and a specific heat capacity of 0.86 kJ·kg^−1^·K^−1^. The heat conductivity is relatively low with a value of 2.7 W·m^−1^·K^−1^. The compatibility of Cofalit^®^ with Solar Salt and Hitec XL has been investigated by Calvet [26]. The maximum operation temperature of the isothermal test was 500 °C during a test duration of 500 h. In Solar Salt the Cofalit^®^ ceramic is stable at the chosen steady state conditions. However its compatibility still requires investigations at dynamic thermo-chemical conditions. In the heat transfer medium HITEC XL, a thin layer consisting of calcium silicate was formed on the surface of the Cofalit^®^ ceramic.

Ortega [27] investigated another industrial waste product as potential filler material in hot air, synthetic oil and molten salt: Two electric arc furnace slags from two different steel manufactures in Spain. The slags are in direct contact with the Solar Salt for 500 h and a maximum operation temperature of 500 °C. No contamination of the molten salt or interaction layers between slag and salt were observed after the thermal treatment [27]. Disadvantages of the industrial waste as filler material are the uncertain availability in future as well as the toxicity and environmental compatibility.

Grirate [28] did investigate granite, basalt, quartzite, marble and hornfels from Morocco. The natural stones were analysed in terms of form, colour, grain size, hardness as well as the presence of carbonate elements. Additionally, physical properties (porosity, density, compressive strength, heat capacity) and the thermal stability up to 400 °C in an air atmosphere have been determined. Quartzite was chosen as the most suitable filler material because of its high thermal conductivity (caused by the high percentage of the mineral quartz) and the high compressive strength and hardness.

Similar investigations of natural stones have been performed by Martin [29]. Basalt, diabas and quartzite were chosen as potential filler materials due to their high density and compressive strength. Initially, the thermal stability up to 900 °C in air and the presence of carbonate elements, using hydrochloric acid, have been determined. Additionally basalt and quartzite were investigated in Solar Salt at isothermal and cyclic conditions up to 560 °C with a maximum operation duration of 1000 h. Furthermore the specific heat capacity of basalt and quartzite was determined. Visual inspection of both basalt and quartzite showed that they are compatible with Solar Salt at high temperatures. The stability was confirmed by thermogravimetry analysis. Further analyses of the mineral content before and after the thermo-chemical oven test and thermal test in Solar Salt with higher test duration are necessary [29].

### Latent heat storage in nitrates

In case of latent energy storage another thermal property needs to be considered, the thermal conductivity *k*. This property gets important because no common heat exchanger can be utilized to assist with the heat transfer. Additionally the density and the enthalpy at the phase transition are important because they determine the volumetric storage capacity. At the phase transition – which occurs within a temperature range of approximately 10 K or less – the change of enthalpy per temperature (∂*H*/∂*T*)_p_ increases considerably.

Therefore the energy stored within a limited temperature range of 10 K is increased by approximately more than one order of a magnitude in phase change materials compared to sensible storage materials. For example a phase transition taking place within 10 K with a melting enthalpy of 150 kJ·kg^−1^ requires a heat capacity of 15 kJ·kg^−1^·K^−1^ to result in an equal storage capacity per 10 K. For KNO_3_ the melting enthalpy was measured to be ≈100 kJ·kg^−1^ [3].

**Figure 10:**
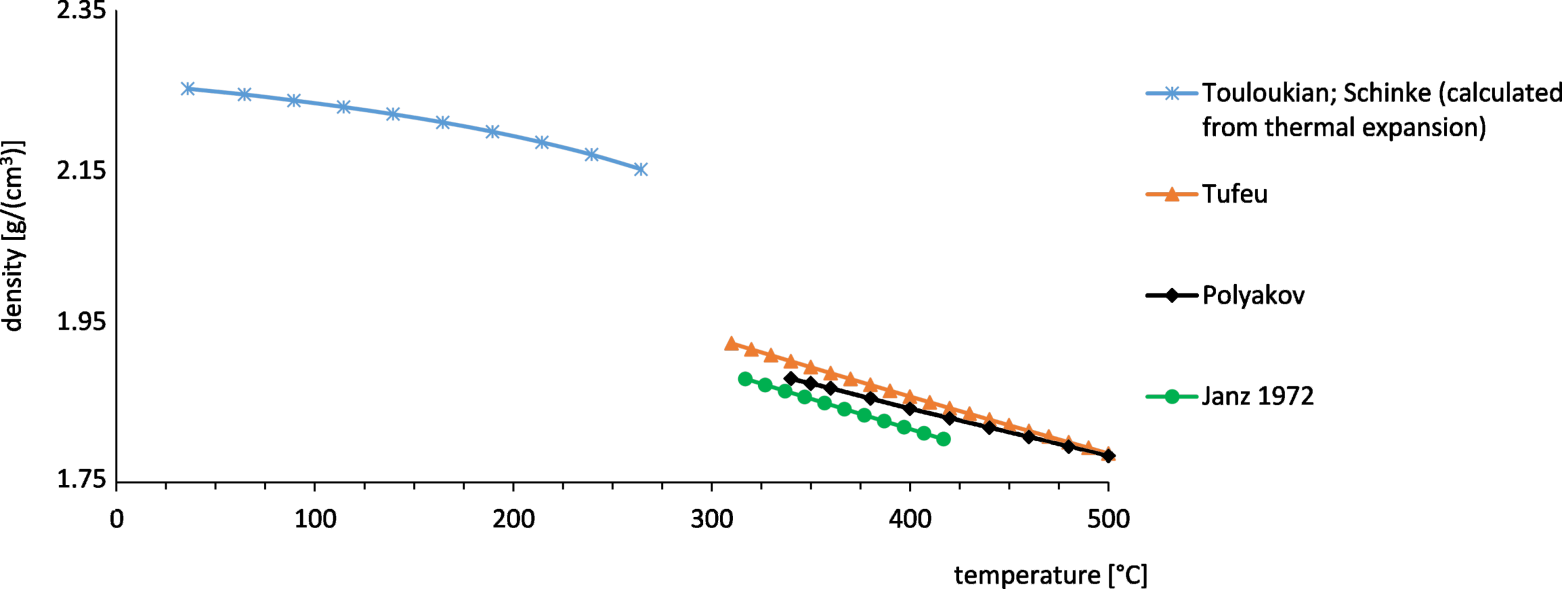
Density of NaNO_3_ [16,30–32].

For the calculation of the volumetric storage capacity the density has to be known as well. In Figure 10 the density of NaNO_3_ is plotted versus the temperature.

**Figure 11:**
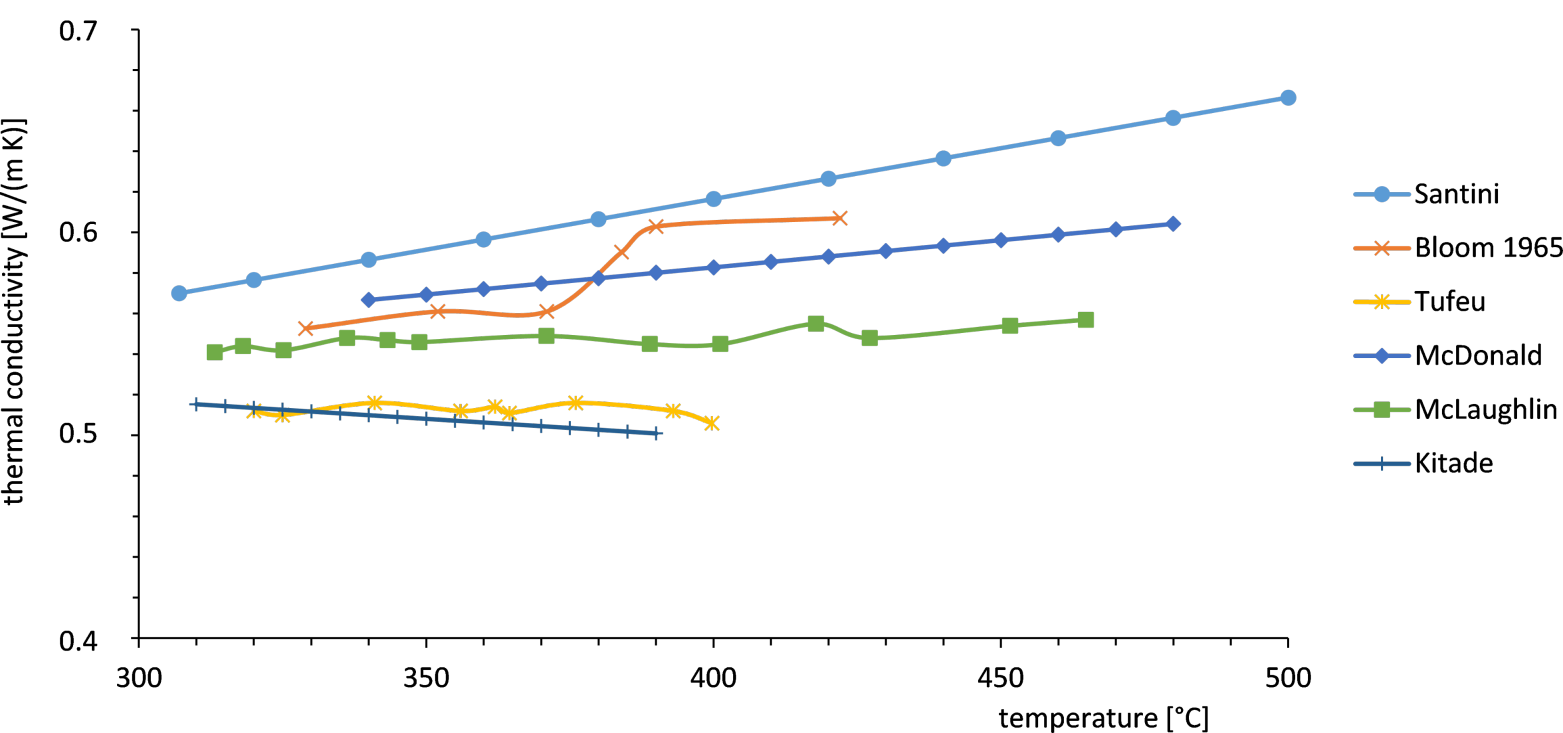
Thermal conductivity of NaNO_3_ in the liquid range [10–11,13–14,31,33].

As was discussed above the thermal conductivity is an important parameter. Therefore consistent data are required. However there is a lack of consistent data for the thermal conductivity as is shown in Figure 11. Further investigations need to be performed to clarify the discrepancy of the values.

### Combination of PCMs with sensible heat storage for effective heat capacity enhancement

Common storage systems are sensible materials or phase change materials. Some research has been performed on the combination of several phase change materials which can result in a sensible storage type system with enhanced effective heat capacity as will be discussed more into detail in the following section.

Sensible storage materials are characterized by the specific heat capacity. The amount of stored sensible heat in storage materials is correlated with the temperature range used and with the specific heat capacity of the storage material. An improved heat storage material could additionally use the enthalpy of fusion of the storage material in order to realize higher effective specific heat capacities. The advantage of combined specific heat capacity of the material and enthalpy of fusion of the phase change process is a higher energy density. A possibility to realise that purpose is the application of serially connected phase change materials with various melting temperatures. Alternatively phase change materials with a melting range as opposed to a melting point can be used.

**Figure 12:**
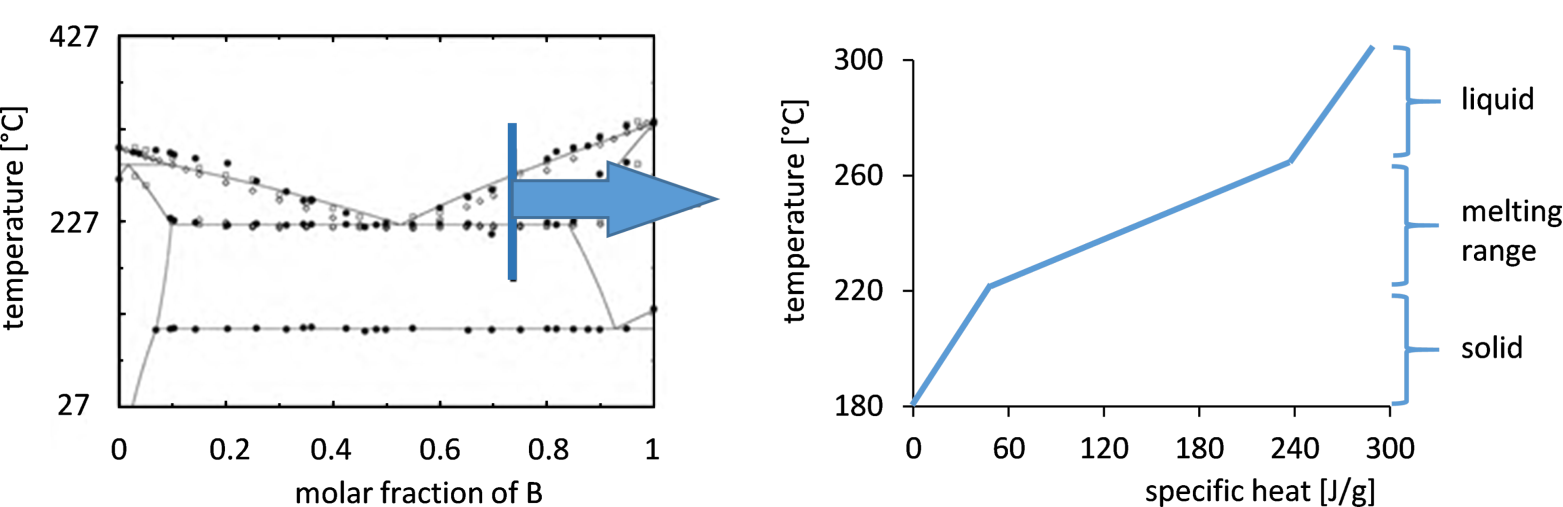
Phase diagram of KNO_3_–NaNO_3_ [34] and the phase dependent enthalpy increase during thermal charging of the selected salt mixture (30 wt % KNO_3_ + 70 wt % NaNO_3_).

Storage materials with melting range can be salt mixtures or alloys. At temperatures below the phase transition solid components are in equilibrium. During the storage process the ratio of molten to solid state increases as well as the temperature. This technique therefore combines sensible and latent heat storage. Figure 12 illustrates the phase diagram of a common salt mixture (KNO_3_ + NaNO_3_) and the temperature characteristic during the charging process for the salt mixture with 30 wt % potassium nitrate and 70 wt % sodium nitrate.

The temperature of the storage material increases during the charging process, which is characteristic for sensible storage materials. The stored heat is used for both melting and heating of the salt. Hence the temperature rise is lower than by a sensible storage material with the same specific heat capacity. The reason is that the effective specific heat capacity of the salt mixture with a melting range is considerably higher as compared to the common molten salt mixtures. The effective average specific heat capacity in the melting range *c*_p,eff_ consists of two terms: the specific heat capacity *c*_p_ in the melting range and the ratio of the melting enthalpy *h* and temperature range *T* of the melting range:


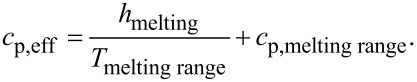


The implementation of salt mixtures with melting range in effective processes requires the uniform distribution of the enthalpy of fusion in the melting range which is influenced by the miscibility of the salt mixture in the solid state [35]. Binary salt mixtures can be also classified on the basis of its miscibility in the solid state. In the liquid state most salt mixtures are completely miscible [36]. Various types of binary phase diagrams do exist [36–37]:

- Complete miscibility in the liquid and solid state with or without minimum melting point (continuous solid solution)
- Complete miscibility in the liquid state and insolubility in the solid state (simple eutectic system)
- Complete miscibility in the liquid state and partial miscibility in the solid state
  a) Segregation by eutectic reaction (eutectic system with limited solid solubility)
  b) Segregation by peritectic reaction
- System with intermetallic phases
  a) Congruently melting compounds
  b) Incongruently melting compounds

Based on the data of Martin [35,38], binary mixtures with complete and partial miscibility in the solid state are suitable as heat storage material with a melting range. Peritectic reactions are diffusion-controlled, so that the reaction can be inhibited by fast heating or cooling rates. As a result of the inhibited peritectic reaction, the composition of the solid salt and the melting characteristic can change.

**Figure 13:**
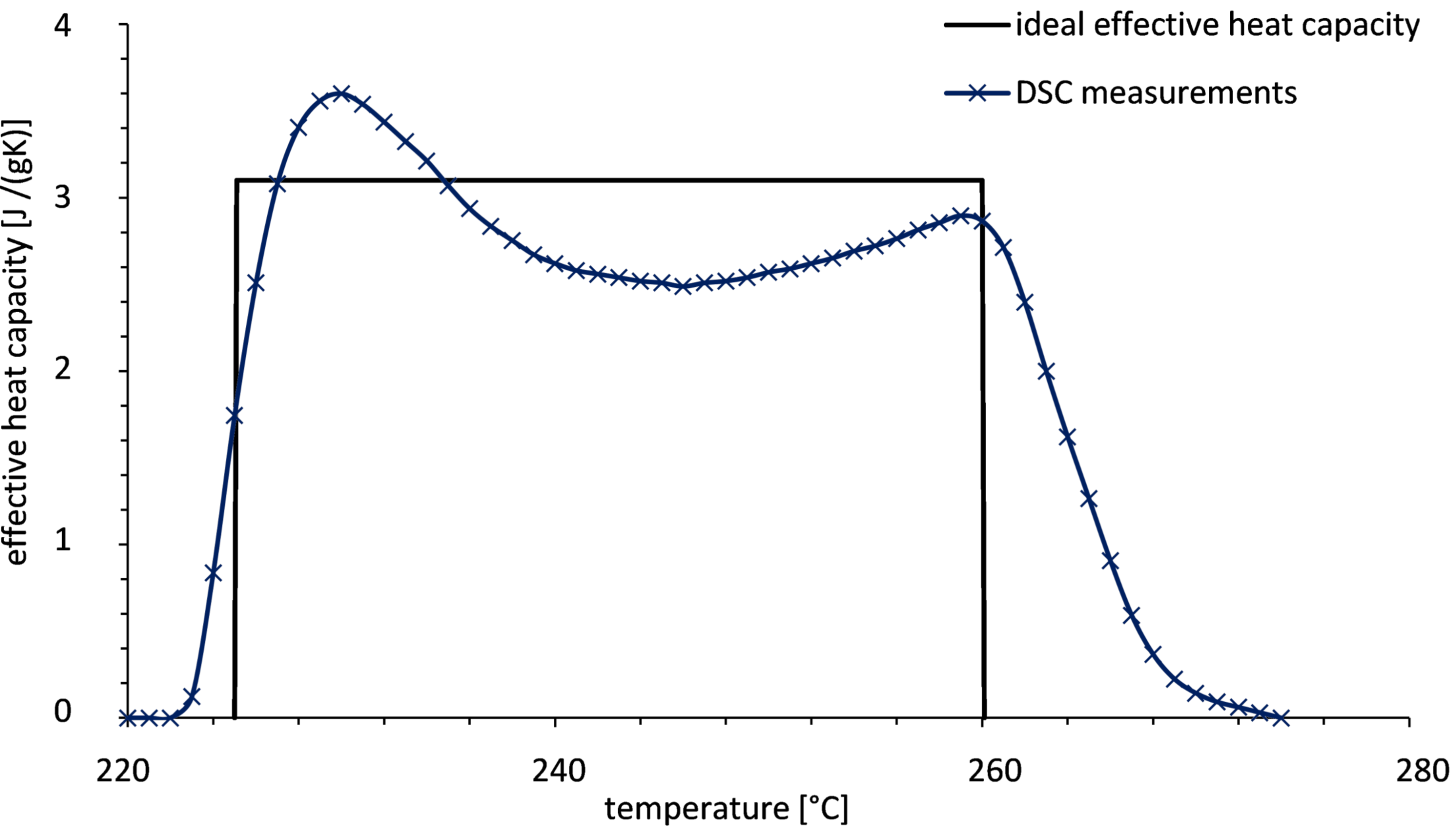
Specific enthalpy of fusion for the salt mixture KNO_3_–NaNO_3_ [35,38].

In the studies of the mixture of 30 wt % potassium nitrate (KNO_3_) and 70 wt % sodium nitrate (NaNO_3_) the investigations focused on the distribution of the enthalpy of fusion as shown in Figure 13. The salt mixture has an enthalpy of fusion of 120 kJ·kg^−1^ that is distributed uniformly in a melting range from about 222 to 260 °C.

In the lab-scale storage unit an effective specific heat capacity of about 3 kJ·kg^−1^·K^−1^ can be achieved. Thus the specific heat capacity of the salt mixture is doubled at least by using a salt mixture with melting range. Thus the use of salt mixtures with a melting range is an interesting option to increase the thermal energy density of sensible storage materials.

## Conclusion

This chapter presented various types of thermal energy storage materials and concepts. At the time of writing, in the field of concentrated power applications (CSP), molten nitrate salts (predominantly a mixture of 60 wt % NaNO_3_ and 40% KNO_3_, so called “Solar Salt”) are used exclusively. Concerning the thermal properties of these salts, reliable data of single salts are available. However, salt mixtures consisting of ions different from sodium and potassium have to be investigated further in the future. Those multicomponent salt mixtures feature much lower melting points compared to Solar Salt and could be attractive materials for direct thermal energy storage for CSP applications.

With regard to the thermal decomposition, the investigation is complex. Many factors influence decomposition reactions, such as the type of the salt, the temperature and the gas phase composition. There is still a need to examine thermal decomposition processes of nitrate salts.

Research also has been performed on storage materials with a melting range with the aim to increase the effective average specific heat capacity. The concept was demonstrated with a mixture of 30 wt % KNO_3_ and 70 wt % NaNO_3_. However there is still a need to examine the handling of the salt and the cyclic stability.

In order to reduce costs of sensible energy storage materials, molten salts are partially replaced by filler materials. It was shown that concrete and mortars experienced softening when thermally cycled in molten salts and therefore are less suitable. In contrast, basalt and quartzite look promising from the first experiments, but long-term stability measurements are still required.
